# *In vivo* analysis of the *Escherichia coli* ultrastructure by small-angle scattering

**DOI:** 10.1107/S2052252517013008

**Published:** 2017-09-26

**Authors:** Enrico F. Semeraro, Juliette M. Devos, Lionel Porcar, V. Trevor Forsyth, Theyencheri Narayanan

**Affiliations:** a ESRF – The European Synchrotron, 38043 Grenoble, France; b Institut Laue–Langevin, 38042 Grenoble, France; c Life Sciences Department, Keele University, Staffordshire ST5 5BG, England

**Keywords:** *in vivo* analysis, *Escherichia coli*, ultrastructure, small-angle scattering

## Abstract

The flagellated Gram-negative bacterium *Escherichia coli* is one of the most studied microorganisms. Despite extensive studies as a model prokaryotic cell, the ultrastructure of the cell envelope at the nanometre scale has not been fully elucidated. Here, a detailed structural analysis of the bacterium using a combination of small-angle X-ray and neutron scattering (SAXS and SANS, respectively) and ultra-SAXS (USAXS) methods is presented. A multiscale structural model has been derived by incorporating well established concepts in soft-matter science such as a core-shell colloid for the cell body, a multilayer membrane for the cell wall and self-avoiding polymer chains for the flagella. The structure of the cell envelope was resolved by constraining the model by five different contrasts from SAXS, and SANS at three contrast match points and full contrast. This allowed the determination of the membrane electron-density profile and the inter-membrane distances on a quantitative scale. The combination of USAXS and SAXS covers size scales from micrometres down to nanometres, enabling the structural elucidation of cells from the overall geometry down to organelles, thereby providing a powerful method for a non-invasive investigation of the ultrastructure. This approach may be applied for probing *in vivo* the effect of detergents, antibiotics and antimicrobial peptides on the bacterial cell wall.

## Abstract 2

A multiscale *in vivo* ultrastucture of the Gram-negative bacterium *E. coli* has been derived using combined small-angle and ultra-small-angle X-ray and contrast-variation small-angle neutron scattering methods.

## 1. Introduction   

*Escherichia coli* is a model prokaryotic cell classified as a Gram-negative bacterium whose morphology has been studied over the last 60 years (Lieb *et al.*, 1955 ▸) using different techniques including optical microscopy (Latimer, 1979 ▸), light scattering (Wyatt, 1970 ▸), atomic force microscopy (Lonergan *et al.*, 2014 ▸) and X-ray imaging (Miao *et al.*, 2003 ▸). The inner structure of the cell on the nanometre scale is called the *ultrastructure*, which cannot be resolved by optical microscopy, and transmission electron microscopy (TEM) has remained the only suitable method available until now (Hobot *et al.*, 1984 ▸; Graham *et al.*, 1991 ▸; Beveridge, 1999 ▸; Matias *et al.*, 2003 ▸). While the whole geometry of the bacterium is well known, the complexity of the cell makes any attempt to investigate the ultrastructure a challenge. Indeed, cryo-TEM imaging needs to be performed on thin cell sections obtained after high-pressure freezing and cryosectioning (Matias *et al.*, 2003 ▸) or other more invasive methods (Hobot *et al.*, 1984 ▸) that may introduce artefacts. Nevertheless, progress in these methods has allowed a better understanding of the bacterial ultrastructure (Milne & Subramaniam, 2009 ▸), in particular shedding light on the spatial arrangement within the cell envelope.

Small-angle scattering (SAS) techniques, such as X-ray and neutron scattering (SAXS and SANS, respectively), are unique tools for elucidating the morphology and the internal structure of lipid vesicles and membranes (Kučerka *et al.*, 2008 ▸). The scattered intensity, *I*(*q*), as a function of the modulus of the scattering vector, *q*, is directly related to the Fourier transform of electron/atom density distribution within the object. *q* is given by *q* = (4π/λ)sin(θ/2), where λ is the wavelength of the incident beam and θ is the scattering angle. Therefore, a scattering curve covering a *q* range contains convoluted structural information over a nominal size scale defined by the minimum and maximum values of 2π/*q*.

Thanks to the recent advances in X-ray instrumentation, it is now possible to perform combined SAXS and ultra-SAXS (USAXS) measurements on low-contrast samples such as bacteria and exploit the wide *q* range available, ∼0.001–6 nm^−1^ (Narayanan *et al.*, 2017 ▸). The SAXS/USAXS combination enables the probing of structural features from several micrometres down to a nanometre that may be used to complement cryo-TEM observations. This size range covers a full description of the bacterium, providing a partial overlap with conventional techniques such as optical microscopy and down to the scale of the ultrastructure. Compared with cryo-TEM, (U)SAXS and SANS measurements can be performed *in vivo*, but the challenge here involves the deconvolution of all of the structural features contained in the scattering curve. For example, SANS has been employed to follow the changes in thylakoid cell membrane periodicity upon illumination by light (Liberton *et al.*, 2013 ▸; Nagy *et al.*, 2014 ▸). Similarly, SAXS and USAXS have been used for screening antibiotic effects on the cell structure in *E. coli* suspensions (von Gundlach *et al.*, 2016 ▸). However, the analysis has so far remained at a qualitative level.

In this paper, we present a method for multiscale structural analysis of the *E. coli* bacterium with the aim of providing a framework for quantitative structural elucidation of any diderm prokaryotic cells. The presented model spans from the whole micrometre-sized cell down to its ultrastructure following a hierarchical description. This was obtained by analyzing combined USAXS/SAXS *in vivo* data constrained by contrast-variation SANS data at three match points and full contrast. The model formalism includes colloidal cell-body, multilamellar membrane cell-envelope and polymer-like flagella features. This allowed the quantitative determination of the cell-envelope electron density and inter-membrane distances.

## 2. Modelling a diderm bacterium   

The formulation of a scattering model for a cell requires some knowledge of its structure and composition. These are essential to obtain an estimation of the typical size, which will be related to a specific *q* range, alongside the volume, *V*, and average scattering length density (SLD), ρ, of each component. These estimations are necessary to understand how a specific component or area of the bacterium is visible or influences the measured scattering curve. The leading term of scattering intensity is proportional to these quantities *via* the relation *I*(*q*) ∝ (*V*Δρ)^2^, where Δρ is the difference between the SLDs of the particular scatterer and the surrounding medium. Using the information available in the literature, possible volume ratios with respect to the cell body and estimations of both X-ray and neutron SLDs (XSLD and NSLD, respectively) are listed in Supplementary Tables S1 and S2, respectively.

### 2.1. Colloidal model for the cell body   

The *E. coli* cell body is rod-shaped, with a diameter of 0.4–0.8 µm and a length of 1–4 µm depending on the strain and the growth phase (Maclean & Munson, 1961 ▸; Chien *et al.*, 2012 ▸). In common with most prokaryotic cells, *E. coli* has neither a nucleus nor organelles. The interior of the cell, the cytoplasm (CP), is a dense, crowded dispersion of macromolecules. The nucleoid region comprises the main DNA ring, which is tightly folded, and proteins. The DNA strand may have a total length of up to 1 mm but it occupies a tiny fraction of the total volume of the cell (up to ∼0.6%) and therefore its contribution to the total scattering intensity is not expected to be significant. The non-nucleoid region of the cytoplasm is a concentrated solution of macromolecules, primarily consisting of proteins and ribosomes, which together can occupy up to around 30% of the available volume (Zimmerman & Trach, 1991 ▸). In this region the largest objects are the ribosomes, which have a diameter *d* of ∼20 nm.

The USAXS setup allowed investigation of the micrometre- and submicrometre-sized features, providing measurements with high-quality data for *q* values down to 2 × 10^−3^ nm^−1^. In this range, *I*(*q*) can be modelled in terms of a colloidal form factor, specifically involving end-capped cylinders (Kaya & Souza, 2004 ▸) as shown in Supplementary Fig. S1. To avoid numerical artefacts in the oscillations at high *q* values (*qR* ≫ 1) for cylindrical-like form factors, the shape of the cell was approximated by an ellipsoidal core, corresponding to a scattering amplitude *A*(*q*) (Pedersen, 1997 ▸), 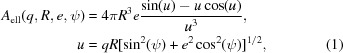
where *R* and *e* are the minor radius and aspect ratio, respectively, and ψ is the polar angle in spherical coordinates which describes all of the possible orientations of suspended cells. The USAXS *q* range primarily concerns size scales from a few hundreds of nanometres to several micrometres, and therefore features arising from the cytoplasm content (such as the ribosome), the ultrastructure or the flagellar radius are not visible. In this *q* range, the *E. coli* scattering is dominated by the entire cell body enclosing the cytoplasm representing the core (Supplementary Fig. S1). The contribution of the bacterial capsule is not explicitly included since it consists of a very diffuse envelope primarily made of polysaccharides (Whitfield & Roberts, 1999 ▸; Parmar *et al.*, 2014 ▸) with very low contrast.

### 2.2. Membrane model for the cell envelope   

**Figure 1:**
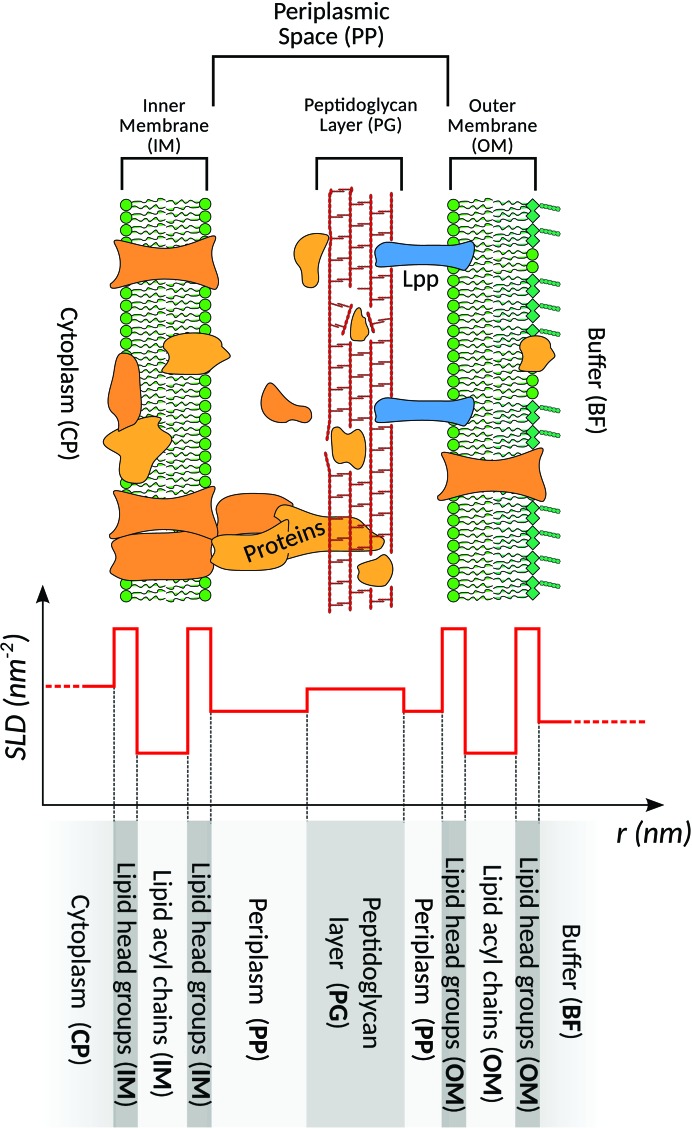
Top: schematic diagram of the *E. coli* ultrastructure. The diderm cell envelope is distinguished by the presence of the periplasmic space (PP), which is separated from the cytoplasm (CP) by the inner membrane (IM). In turn, the periplasm is separated from the outside by the outer membrane (OM), which is firmly bound to the peptidoglycan layer (PG) inside *via* Lpp proteins. Bottom: scheme of the core multiple shell SLD profile used to model the bacterial scattering form factor.

*E. coli* is a diderm cell classified as a Gram-negative bacterium, hence the cell envelope is characterized by two phospholipid membranes, as depicted in Fig. 1 ▸. The inner membrane (IM) consists of phospholipids (*e.g.* phosphatidyl­ethanolamine and phosphatidylglycerol) comprising mostly palmitic acids (Kaneshiro & Marr, 1961 ▸; Cronan, 1968 ▸; De Siervo, 1969 ▸; Oursel *et al.*, 2007 ▸). The IM contains membrane proteins that perform most of the functions of the cell, while the outer membrane (OM) acts as a protective barrier. The OM is asymmetric, with a similar phospholipid content to the IM in the inner leaflet, but with a high concentration of lipopolysaccharides (LPS) in the outer layer. The protein content in the OM is thought to be lower than that in the IM, and it is only decorated by membrane proteins responsible for transport and a few enzymes such as protease and phospholipase (Silhavy *et al.*, 2010 ▸).

The region enclosed by the IM and OM is called the periplasm (PP). It is a highly oxidizing environment that is less dense in protein than the cytoplasm and serves to trap potentially dangerous enzymes to the cell. A portion of the periplasmic space is occupied by the peptidoglycan layer (PG). This is supposed to be a porous and stiff net-like structure that defines the shape of the cell envelope and prevents structural damage, for example by osmotic pressure. The PG consists of disaccharide chains cross-linked by four-unit amino-acid chains (Zaritsky & Helmstetter, 1992 ▸; Pink *et al.*, 2000 ▸; Gan *et al.*, 2008 ▸). It is linked to the OM by Braun’s lipoproteins (Lpp), the length of which is about 8.3 nm (Shu *et al.*, 2000 ▸). These are covalently bound to the PG at one extremity, while the other is embedded in the OM.

The cell envelope occupies up to 20% of the total volume, therefore the total mass of the lipid content of the membranes could contribute significantly to *I*(*q*). As the shell thickness is less than ∼35 nm, considering two membranes of ∼5 nm each and a periplasmic width of around 10–25 nm (Graham *et al.*, 1991 ▸) the ultrastructure should contribute at higher *q* values compared with the USAXS range. The cytoplasmic core model is completed by several shells: a cytoplasmic ellipsoidal core with uniform density is surrounded by a series of layers describing the structure of the diderm cell, as illustrated in Fig. 1 ▸. Each layer is represented by an ellipsoidal shell of uniform average density with a homogeneous average XSLD or NSLD. The membrane model is based on the state-of-the-art cryo-TEM observations (Matias *et al.*, 2003 ▸) on thin sections of vitrified bacteria, including *E. coli* K-12 and *Pseudomonas aeruginosa*. Therefore, in principle this model can be used to describe other diderm bacteria. The core multiple shells form factor is expressed as (Pedersen, 1997 ▸)
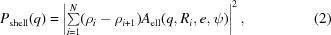
where ρ_*N*+1_ = ρ_buffer_, *A*_ell_(*q*, *R_i_*, *e*, ψ) is the scattering amplitude of an ellipsoid (see equation 1) with minor radius *R_i_* and scattering length density ρ_*i*_ (the *R*_1_ and ρ_1_ values define the core). Note that the width of each shell *R*_*i*+1_ − *R_i_* is constant over the entire surface, as the aspect ratio *e* is only applied to the radius of the cell body, *R*_1_.

### 2.3. Polymer model of the flagella   

Each bacterium possesses up to ten flagella, which in turn are anchored to the cell by a protein complex that crosses the entire cell envelope. A single flagellum is a very long (up to 15 µm) cylindrical macromolecular assembly with flagellin subunits (Asakura *et al.*, 1964 ▸; Yamashita *et al.*, 1998 ▸). Their radius is ∼10 nm (Yamashita *et al.*, 1998 ▸) and each flagellum describes a helix, the coil length of which changes depending on the cell motion (Calladine, 1978 ▸; Turner *et al.*, 2010 ▸). Owing to their length, the volume ratio between the flagella and the cell body is 0.2–8%. Hence, their scattering contribution might be negligible as for DNA, but in the extreme case may be comparable to that of the cell membrane.

Along with the membrane model, flagella can be described in terms of the self-avoiding walk (SAW) model of polymer chains. This representation is appropriate for flagella since their function is to rotate and self-propel the cell body, and a severe entanglement of these long filaments would not lead to any motion. The radius of gyration, *R*_g_, of a SAW polymer with contour length *L* and *N* repeating blocks of repetition length *b* = *L*/*N* is given by (Flory, 1969 ▸)
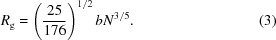
Considering short and wavy filaments (*L*/*b* = 2000/20), the smallest *R*_g_ value is estmated to be ∼110 nm. Therefore, even the shortest flagella should scatter by an asymptotic power law in the *q* region of the cell envelope, whereas their contribution at smaller *q* values is orders of magnitude below the cell body, 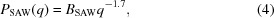
where *B*_SAW_ ∝ (ρ_SAW_ − ρ_buffer_)^2^. The value ρ_SAW_ is expected to be very close to that for proteins, as the flagellum is a purely protein-based assembly.

### 2.4. Multiscale model   

The model including cell body, cell envelope and flagella is given by the equation 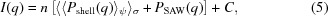
where *n* is the number density of cells and *C* is a constant background to account the scattering at high *q* from unidenti­fied contributions. The cross-term of the cell and flagella scattering functions is neglected since the flagella contribution is only significant in the asymptotic power-law region. The first angular brackets are related to the orientation average 〈*f*(*x*)〉_ψ_ = 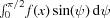. The second pair represents the cell size and periplasmic width polydispersities (Trueba & Woldringh, 1980 ▸), which have been included with a normal distribution *D*(*R*) centred on a mean value 〈*X*〉, with a standard deviation σ, 〈*f*(*x*)〉_σ_ = 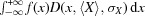. Both σ values are not meant to give a precise polydispersity or detect shape fluctuations; they are rather used as smearing functions based on real characteristics of the cell.

## 3. Experimental methods   

### 3.1. Sample preparation   

One Shot TOP10 chemically competent *E. coli* cells from Invitrogen (K-12 strain, similar to the DH10B strain) were used in this study. Colonies were grown in LB medium (Sigma–Aldrich) with ampicillin (100 µg ml^−1^, Euromedex) at 37°C to an OD_600_ of ∼1 (∼8 × 10^8^ cells ml^−1^). The cells were centrifuged (1000*g*, 4°C), washed and gently resuspended in nutrient-free and sterile-filtered phosphate-buffered saline (PBS) pH ∼7.4 to an OD_600_ of ∼10 for SAS experiments. PBS and deuterated PBS (D-PBS) were adjusted to pH ∼7.4 and pD ∼7.4, respectively. Contrast-matching measurements were carried out on bacteria resuspended in D-PBS or in various ratios of PBS and D-PBS (further details are provided in the Supporting Information).

### 3.2. Small-angle scattering   

USAXS/SAXS measurements were performed on the TRUSAXS beamline (ID02) at the ESRF. The instrument uses a monochromatic beam with a wavelength λ of 0.0995 nm collimated in a pinhole configuration. Measurements were performed at room temperature with sample-to-detector distances of 30.8, 10.0 and 1.0 m covering a *q* range of 0.002–7 nm^−1^. A Rayonix MX170 detector was used for these measurements. The flux of the incident X-ray beam was less than ∼2 × 10^12^ photons s^−1^. Samples were contained in quartz capillaries with a diameter of ∼1.8 mm and a wall thickness of ∼0.01 mm. The measured two-dimensional scattering patterns were normalized to absolute scale after instrument-specific corrections and were azimuthally averaged to obtain the corresponding one-dimensional SAXS/USAXS profiles. The normalized cumulative background from the buffer, sample cell and instrument were subtracted to obtain the *I*(*q*). SANS measurements were performed at the large dynamic range SANS instrument D22 at the ILL. This instrument also employs pinhole collimation and a monochromatic beam. The experiments were performed with λ = 0.6 nm (Δλ/λ ≃ 0.1) using three sample-to-detector distances, 17.6, 5.6 and 1.4 m, covering a *q* range of 0.02–3 nm^−1^. Samples were contained in quartz Hellma cuvettes with sample thickness 1 mm. The two-dimensional SANS data were reduced using a similar procedure as described above for the SAXS data.

## 4. Results and discussion   

Considering the limited scattering features of bacteria, a genuine model (equation 5) must be as simple as possible and contain the smallest number of parameters. Using a more complex membrane model (Kiselev *et al.*, 2002 ▸; Foster, 2011 ▸) would increase the number of parameters and may lead to false-positive results. A minimal multiscale form-factor model was used to fit the combined USAXS/SAXS and contrast-variation SANS intensities. The latter were used to add more constraints during the fitting procedure. For this purpose, five different buffers were used: PBS and D-PBS to have two references at 0 and 100% in D_2_O weight ratio and then three different mixtures with D_2_O contents of 65 wt% to match the DNA/RNA contributions, of 42 wt% to equal the average NSLD of proteins and protein complexes and of 11 wt% to match the scattering signal from the phospholipid membranes.

To make a realistic fit, each parameter needs strict boundary conditions and a self-consistency check must be performed on the obtained results. Fitting SAS data with membrane models, where the parameters have a high degree of correlation, is usually hard because of the huge number of local minima in the χ^2^ function. In this work, fits were performed using a genetic selection algorithm (Heftberger *et al.*, 2014 ▸), which is particularly suitable for such minimization as it is designed to avoid false convergences in local minima.

**Figure 2:**
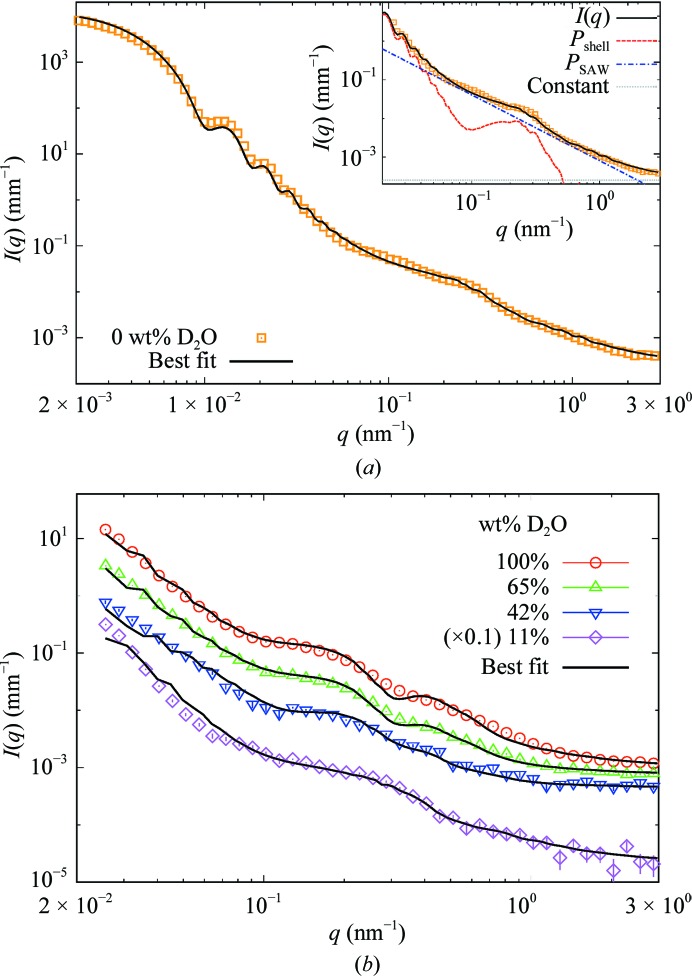
(*a*) Representative USAXS/SAXS from an *E. coli* suspension and the corresponding fit with (5). Data are for the sample at 0 wt% D_2_O in suspension medium with OD_600_ = 10. The sum of the membrane model 〈*P*_shell_(*q*)〉, the SAW polymer model *P*_SAW_(*q*) and the constant value is shown in the inset. (*b*) SANS data at 11, 42, 65 and 100 wt% D_2_O were fitted with (5).

Best fits are shown in Fig. 2 ▸ and the corresponding parameters are tabulated in Table 1 ▸. Both USAXS/SAXS and SANS data were fitted with a single model accounting for global and local parameters. The complete set of values is able to represent the entire model by meaningful values giving an optimum cumulative χ^2^ and to fulfill self-consistency criteria. In the context of this analysis, a set of results is self-consistent if it is able to describe the model in its entirety, including features that are not explicitly incorporated in equation (5).

**Figure 3:**
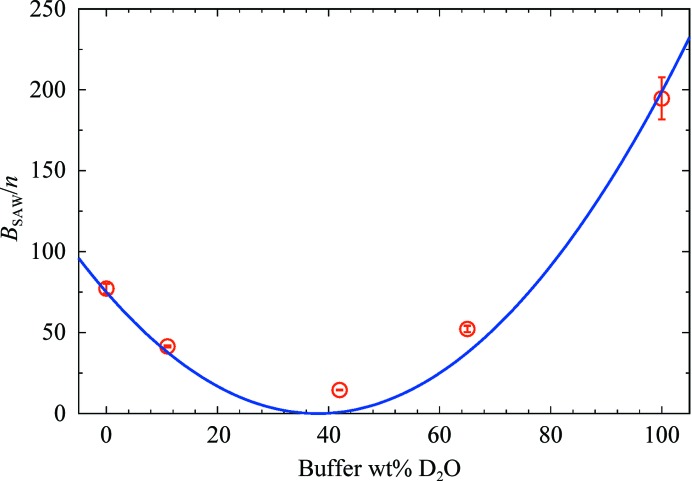
Fitting of *B*_SAW_/*n* values as a function of the nominal D_2_O concentration. The minimum of the parabola is close to the matching point for proteins, which is roughly 36 wt% D_2_O (1.9 × 10^−4^ nm^−2^).

(i) Values of ρ_CP_, ρ_PP_, ρ_PG_ and ρ_ME_ from SANS measurements are expected to be linear with the D_2_O content in the buffer, which in turn scales linearly with ρ_BF_, because of the semi-permeability of membranes (ρ_ME_ accounts for the average SLD of the four lipid head-group layers). The mixture of water and D_2_O is free to diffuse through the periplasm into the cytoplasm. For the same reason, D_2_O concentration is also in equilibrium with the hydration water of the lipid head groups in both the inner and outer membranes (Supplementary Fig. S2).
(ii) Once the linearity criterion for ρ_CP_, ρ_PP_, ρ_PG_ and ρ_ME_ obtained from SANS curves at D_2_O concentrations of 11, 42, 64 and 100% is fulfilled, corresponding values for the curve at 0% D_2_O can be extrapolated with precision. These values were then used to fit the corresponding SANS data, leaving only *n*, *B*_SAW_ and *C* as free parameters (Supplementary Fig. S3).
(iii) From the last fit, *n* and *B*_SAW_ parameters are obtained and used as a further control. The six *n* values obtained from the fits are expected to be the same based on the confidence in sample preparation. Indeed, they are comparable and give an average of 〈*n*〉 = (6.8 ± 0.6) × 10^9^ ml^−1^. The sample concentration is a prefactor in the *I*(*q*), hence the square root of the relative error of *n*, ±9%, can be considered as a maximum global error on each XSLD/NSLD profile.
(iv) Finally, as a last test of self-consistency, the contribution of the SAW polymer was verified. Flagella content is purely protein-based, therefore *B*_SAW_ values, normalized by *n*, are expected to have a quadratic dependence on the buffer NSLD, following the equation *B*_SAW_ ∝ *n*(ρ_SAW_ − ρ_BF_)^2^. The fit is shown in Fig. 3 ▸, giving a match point at 38.0 ± 1.7 wt% D_2_O, equivalent to an NSLD value of (2.08 ± 0.12) × 10^−4^ nm^−2^, and is consistent with the expected value (1.9 × 10^−4^ nm^−2^) for proteins. A similar test for the consistency of the SAW term can be performed from USAXS/SAXS data in terms of the Ornstein–Zernike (OZ) Lorentzian structure factor. Considering two extreme *R*_g_ for flagella, short/wavy (*L*/*b* = 2000/20) and long/smooth (*L*/*b* = 15000/500) flagella, with *R*_g_ = 113 nm and *R*_g_ = 1393 nm, respectively, the fitted *P*_SAW_(*q*) is consistent with the asymptotic trends of simulated OZ curves (Supplementary Fig. S4). A contribution to this term from the capsule of the cell cannot be excluded, since it is also composed of long polysaccharide chains (Whitfield & Roberts, 1999 ▸).

**Figure 4:**
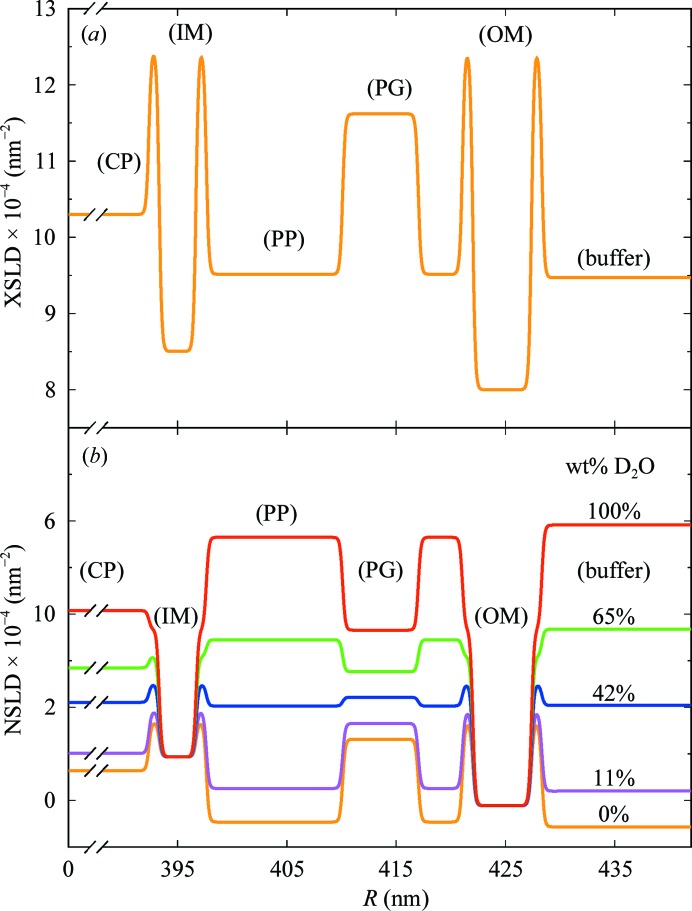
(*a*) XSLD profile for the system suspended in PBS buffer. (*b*) NSLD profiles for five different contrast-match points. The *x* axis refers to the minor radius of the ellipsoid. The same profile is applied all over the ellipsoid. The smearing of the rectangular SLD profile is only used for better visibility.

Both X-ray and neutron SLD profiles of the cell envelope are displayed in Fig. 4 ▸. The centre-to-centre distance between the IM and OM, *C*_OM_, is the key variable for SANS data from 42 to 100 wt%, where both IM and OM acyl-chain layer SLDs, ρ_TI_ and ρ_TO_, appear as two deep wells over a high SLD profile. This width dominates over other features of the SLD profile, and delineates the position of the maxima in the corresponding scattering curves. The resulting effective average width of the periplasmic space is 23 nm, which is in perfect agreement with the expected range of 11–25 nm (Graham *et al.*, 1991 ▸; Matias *et al.*, 2003 ▸). The centre-to-centre distance between the PG and OM, *C*_PG_, is fundamental to the shift observed in the maximum in SANS data at 11 wt%. This feature at *q* ≃ 0.27 nm^−1^, which is also observed in SAXS data, results from a combination of three high-contrast layers, *i.e.* the two membranes and the peptidoglycan region. *C*_PG_ is 11 nm, which is also consistent with the length of the cylindrical Braun’s lipoprotein (Lpp-56; Shu *et al.*, 2000 ▸). The presence of such a scattering feature suggests a low-contrast periplasm which gives visibility to the peptidoglycan layer. This is in contradiction with the idea of a ‘periplasmic gel’, concept that was derived from the cryo-TEM observations on frozen and chemically fixed bacteria (Beveridge, 1999 ▸), where staining compounds may have biased the determination of the effective electron densities. Instead, it is in agreement with the observation of Matias and coworkers where the experimental design minimized the alterations of the samples (Matias *et al.*, 2003 ▸). The core radius, *C*_IM_, and aspect ratio, *e*, are entirely obtained from the USAXS data at low *q*. They describe the extension of the cytoplasm, which is linked to the ‘weight’ of the cytoplasmic SLD in the core/envelope structure. The two intra-membrane distances (namely, the centre-to-centre distance of the head groups), *D*_IM_ and *D*_OM_, together with the average width of the lipid head groups, *W*_ME_, represent the total widths of the inner and outer membranes. These parameters are physical but could be strongly correlated with ρ_ME_, ρ_PG_, ρ_TI_ and ρ_TO_. *D*_IM_ and *D*_OM_ values cannot be ascribed to visible scattering features (expected around *q* ≃ 1 nm^−1^), therefore they cannot be identified to precise intra-membrane distances. However, they represent the widths of the acyl-chain regions centred at *C*_IM_ and *C*_OM_, so they are essential for the presence of the oscillation at *q* ≃ 0.18 nm^−1^. A realistic error for *D*_IM_, *D*_OM_, *W*_PG_ (width of PG) and *W*_ME_ is ±1 nm.

A rough estimation of the confidence for the whole set of SLD values can be performed by comparing the XSLD and NSLD profiles at 0 wt% D_2_O content. It is possible to recover the trend of the volume fraction of the hydration water *x*_w_ (or of the protein content *x*_p_) in the regions of interest by using the relation 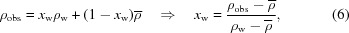
where ρ_obs_ is the measured SLD of the layer, 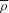 is an approximate theoretical SLD and ρ_w_ is the SLD of water. The aim is to compare *x*_w_ (or *x*_p_) values from the XSLD and NSLD profiles for each layer and extract an estimation of the errors from the discrepancies (Supplementary Table S3).

## 5. Conclusion   

In this article, a multiscale modelling of *E. coli* is presented. The combination of USAXS/SAXS and contrast-variation SANS measurements elucidates the overall geometry of the whole micrometre-sized body and the details of the cell envelope on the nanometre scale. The comparison between SAXS and SANS measurements allowed the mutual exclusion of both X-ray radiation damage and toxic effects owing to the D_2_O medium. A global model was formulated by combining core-shell colloidal, lipid-membrane and polymer-chain formalisms to describe the cell body, the cell envelope and the flagella, respectively. The set of results is self-consistent and is in agreement with the more recent cryo-TEM observations. The global analysis permitted the determination of the membrane electron-density profile and the inter-membrane distances on a quantitative scale. The results reveal a very dilute periplasm, with a dense protein content trapped in (or closely interacting with) the peptidoglycan layer.

To conclude, the synergy of X-ray and neutron SAS techniques can be used as a non-invasive method for the *in vivo* study of the morphology and ultrastructure of Gram-negative bacteria. This offers a great opportunity for applied research on the mechanism of action of antibiotics (Parmar *et al.*, 2014 ▸) and antimicrobial peptides (Matsuzaki, 1999 ▸; Sun *et al.*, 2016 ▸) on cellular membranes by *in vivo* structural analysis.

## Figures and Tables

**Table 1:** Global and local parameters involved in the multiscale model of bacteria (equation 5) including best-fit parameters USAXS values refer to the sample at 0 wt% D_2_O.

					SANS wt% D_2_O				
Parameter		Function		USAXS	0	11	42	65	100
ρ_CP_ × 10^−4^ (nm^−2^)		Average SLD of the CP core		10.26	0.623†	1.01	2.13	2.78	4.08
ρ_PP_ × 10^−4^ (nm^−2^)		Avelage SLD of the PP layer		9.51	−0.38†	0.26	2.04	3.39	5.64
ρ_ME_ × 10^−4^ (nm^−2^)		Average SLD of both the IM and OM head-group layers		12.16	1.69†	1.91	2.49	2.93	3.66
ρ_PG_ × 10^−4^ (nm^−2^)		Average SLD of the PG layer		11.64	1.40†	1.65	2.22	2.75	3.72
ρ_TI_ × 10^−4^ (nm^−2^)		Average SLD of the tail-group layer in the IM		8.56	0.93				
ρ_TO_ × 10^−4^ (nm^−2^)		Average SLD of the tail-group layer in the OM		8.00	−0.11				
ρ_BF_ × 10^−4^ (nm^−2^)		SLD of the buffer solution‡		9.47	−0.56	0.20	2.04	3.64	5.91
*n* × 10^9^ (ml^−1^)		Cell number density		7.4	7.0	7.3	6.2	6.3	6.7
*B*_SAW_ × 10^−11^ (nm^−2.7^)		Intensity factor for SAW polymers		119	54	30.0	9	33	131
Con × 10^−4^ (mm^−1^)		Constant value		3.6	12	2.1	5.0	5.8	9.7
*C*_IM_ (nm)		Mean centre of mass of the IM layer (along the minor radius)		395					
*D*_IM_ (nm)		Centre-to-centre distance of the head-group layers in the IM		4.3					
*C*_OM_ (nm)		Mean centre of mass of the OM layer (distance from *C*_IM_)		29.7					
*D*_OM_ (nm)		Centre-to-centre distance of the head-group layers in the OM		6.3					
*C*_PG_ (nm)		Centre of mass of the PG layer (distance from *C*_OM_)		11.0					
*W*_ME_ (nm)		Width of the head-group layers for both the IM and OM		0.94					
*W*_PG_ (nm)		Width of the PG layer		6.9					
*R*_M_ (nm)		Major radius of the elliptical core (*C*_IM_ × *e*)		910					
σ_CP_ (nm)		Standard deviation of the *C*_IM_ distribution‡		10					
σ_PP_ (nm)		Standard deviation of the *C*_OM_ distribution‡		4					

† Linear extrapolation results.

‡ Fixed parameters.
